# Dimensions of artificial intelligence on family communication

**DOI:** 10.3389/frai.2024.1398960

**Published:** 2024-09-11

**Authors:** Nada Mohammed Alfeir

**Affiliations:** Department of Communication Skills, King AbdulAziz University, Jeddah, Saudi Arabia

**Keywords:** dimensions, AI, privacy, communication, family, society, culture

## Abstract

**Introduction:**

Artificial intelligence (AI) has created a plethora of prospects for communication. The study aims to examine the impacts of AI dimensions on family communication. By investigating the multifaceted effects of AI on family communication, this research aims to provide valuable insights, uncover potential concerns, and offer recommendations for both families and society at large in this digital era.

**Method:**

A convenience sampling technique was adopted to recruit 300 participants.

**Results:**

A linear regression model was measured to examine the impact of AI dimensions which showed a statistically significant effect on accessibility (*p* = 0.001), personalization (*p* = 0.001), and language translation (*p* = 0.016).

**Discussion:**

The findings showed that in terms of accessibility (*p* = 0.006), and language translation (*p* = 0.010), except personalization (*p* = 0.126), there were differences between males and females. However, using multiple AI tools was statistically associated with raising concerns about bias and privacy (*p* = 0.015), safety, and dependence (*p* = 0.049) of parents.

**Conclusion:**

The results showed a lack of knowledge and transparency about the data storage and privacy policy of AI-enabled communication systems. Overall, there was a positive impact of AI dimensions on family communication.

## 1 Introduction

The world is witnessing another historic transition that is different from the other and happening in technology. Dawaesar (2013) has remarked that “Our devices matter to us as much as food and shelter. Technology has altered the flow of time. The overall time that we have for our narrative, our life span, has been increasing, but the smallest measure, the moment has shrunk.” Artificial intelligence (AI) has revolutionized life in every aspect and has brought about unprecedented changes in the ranks and files from businesses to families (Xiao et al., 2021). The term artificial intelligence (AI) refers to building systems and programs that can perform the same functions likewise humans (Boden, 1996). They perform the functions and tasks as humans do. AI has gained traction and prominence in the domain of computer science for decades. They are designed through algorithms where computer vision helps to detect the objective and photos. Natural language processing enables computers to understand the language of humans. Through graphical processing units in the form of chips, computers shape graphics and pictures via mathematical calculations. In addition, the Internet of Things (IoTs) is another form of AI that operates in the form of a network connected with physical devices, sensors, software, and another component of network connectivity in an integrated manner to share data or perform different tasks (Xiao et al., 2021). This initiation has called for renewing the communication infrastructure on newly defined lines with super-heterogenous networks, antennae, and wide bandwidth. Among them, machine learning (ML) is a buzzword as a powerful technology under the auspices of AI due to greater capabilities to be trained continuously to adapt to new changes rapidly (Jiao et al., 2021).

Apart from ML, reconfigurable intelligent surface (RIS) and artificial intelligence (AI) have wider recognition and potential in the entire fabric of wireless communication to enable the sixth-generation networks, aiming to manufacture intelligent, interactive, and collaborative communication environments (Wang et al., 2021). Earlier, intelligence was reckoned as an umbrella character, characterized by the ability of abstract and logical reasoning, and physical as well as emotional intelligence (Sternberg, 2000). Gradually, the concept of intelligence came out from its limited scope and became a unified term for the cognitive capabilities of machines (Robinson, 2014). In technology, intelligence refers to prompt problem-solving with the best automatic solution immediately. Thus, AI has emotional and relational parameters, providing solutions to real-life problems (Schutz, 2014).

Given the vitality of communication, it warrants success for students (Higgins, 2019) and helps teachers achieve their professional objectives through effective communication (Hodis and Hodis, 2021). The way communication flows in a family, and the patterns involved shape a family communication system. Conversation orientation and conformity orientation are the two dimensions of the patterns involved in family communication (Koerner and Mary Anne, 2016). Keeping the relevance of AI for communication, De Togni et al. (2021) explored the transformation in the relationships between humans and machines with the advent of AI. The study pinpointed the need to conceptualize intelligence as an interdisciplinary area of research to draw the theocratical and material distinction between humans and machines, in particular during the communication process (De Togni et al., 2021). The AI emulates the capacity of the human brain for decision-making and skills for problem-solving by utilizing computers and other smart devices. They use several AI-based products such as social bots and virtual agents (Asif and Gouqing, 2024).

Butow and Hoque (2020) have asserted that AI can encode complex human interactions akin to humans with an imitation of emotions as well as the verbal aspects which are part of communication. With the advent of large-scale adoption of AI applications, families make use of it in their communication from voice assistants to navigating within their systems and mobiles.

Druga et al. (2022) scrutinized parents’ roles in developing AI literacies in children. The findings revealed that parents were eager to develop AI literacies among their children. Typically, the focus was on object recognition, voice assistance, image classification, and AI co-design (Druga et al., 2022). Specific dimensions of AI, such as language translation, personalization, and accessibility, influence family communication dynamics in several ways. Language translation capabilities facilitate cross-cultural understanding and inclusivity within multilingual families, enhancing communication effectiveness. Personalization tailors AI interactions to individual family members’ preferences and needs, fostering more personalized and engaging exchanges. Accessibility ensures that all family members, including those with disabilities or different learning styles, can participate fully in communication, promoting inclusiveness and equity within the family unit. These dimensions collectively contribute to shaping more fluid, inclusive, and responsive communication processes within families integrating AI technologies.

### 1.1 Research objectives

The study aims to examine the impacts of AI dimensions on family communication. By investigating the multifaceted effects of AI on family communication, this research aims to provide valuable insights, uncover potential concerns, and offer recommendations for both families and society at large in this digital era. This study employs seven broad dimensions of artificial intelligence, i.e., accessibility, personalization, language translation, privacy, bias, dependence, and safety.

## 2 Literature review

The concept of dimensions in AI refers to the building blocks, characterized by the distinctive attributes or features, encompassing data points in a space with high dimensions. These dimensions are the places for generating AI algorithms, aiming to understand multiple datasets, present in the forms of images and texts, and audio and videos. Following are the operational definition and description of the seven dimensions of AI, employed by the researcher in the present study;

- Accessibility: AI-powered devices and applications can make it easier for family members to communicate with each other, even if they are physically separated. For example, video conferencing software like Zoom or Skype can help families stay connected when they cannot be together in person. Smart home devices like Amazon Echo or Google Home can also be used to make phone calls or send messages hands-free (Dattathrani and De, 2023).
- Personalization: AI can help personalize communication by analyzing data about family members’ preferences, habits, and interests. For example, a messaging app that uses AI could suggest conversation topics based on what family members have talked about in the past. This can help families communicate more effectively and efficiently (Lee and Yoon, 2021).
- Language translation: AI-powered translation software can help families communicate across language barriers. This can be especially helpful in families where members speak different languages or where family members live in different countries (Liao et al., 2023).
- Privacy: AI can potentially compromise family privacy by collecting and analyzing data about family members’ communication habits and behaviors. For example, a smart speaker that is always listening could potentially record private conversations. It is important for families to be aware of the privacy implications of using AI-powered devices and applications and to take steps to protect their privacy (Liu et al., 2022).
- Bias: AI systems can perpetuate biases and stereotypes that exist in society. For example, an AI-powered language translation app may not accurately translate certain languages or dialects, which could reinforce harmful beliefs and attitudes. It is important for families to be aware of these biases and to use AI-powered devices and applications in a way that is inclusive and respectful (Ntoutsi et al., 2020).
- Dependence: Over-reliance on AI-powered devices and applications for communication can potentially weaken in-person communication skills and lead to social isolation. For example, if family members only communicate through texting or messaging apps, they may miss out on the nuances of in-person communication. Families need to balance their use of AI-powered devices and applications with in-person communication (Ntoutsi et al., 2020).
- Safety: AI can help ensure family safety by monitoring for potential risks and threats, such as cyberbullying or online predators. For example, a parental control app that uses AI could monitor a child’s online activity and alert parents to potential risks. However, it is also important to consider the potential for AI-powered surveillance to infringe on privacy and to use these tools responsibly and ethically (Hata et al., 2019).

There is an increasing role of AI in the domain of communication with moderate levels of accuracy, parallel to humans where AI can assume its unconventional role as interlocutor as well as content generator. A practical example of a virtual assistant based on AI is Alexa of Amazon which responds to queries in a human-like manner. Also, there is a growing interest in making embodied AI-enabled robots that are capable of interacting both verbally and nonverbally (Peter and Kühne, 2018). On webpages, AI-enabled chat robots are replacing humans in the form of chatbots; they can exceptionally prepare social media posts for greater engagement and community outreach without any human interaction and intervention (Ferrara et al., 2016). Garg et al. (2022) conducted a comprehensive review of studies about children using communication assistants in their personal and academic communication. According to the review, higher were the concerns of the parents about children using such assistants for their communication stressed the ethical implications of their usage.

On account of a universal perception of technology having dark sides besides all of its prospects such as its addition. Parents feel it challenging to make a balanced habit of their children and vow to regulate a balanced behavior. Thus, they prefer to regulate their children’s usage of mobile and smartphones (Bergert et al., 2020). In the meantime, young children develop the skill of using different electronic gadgets such as smartphones, laptops, and tablets in their early childhood settings. Using the Grounded Theory, research documented that teachers figured out parents’ concerns about their children, using digital devices and technology in their kindergarten classrooms (Schriever, 2021).

Park and Lim (2020) have documented positive results of voice user interface (VUI), such as AI speakers for family cohesion. Wald et al. (2023) examine different types of families, and their motivations for using Virtual assistants (VA) in different ways (parent-only, child-only, or co-use). The study used survey data from 305 Dutch parents with at least one child between the ages of 3 and 8, who had a Google Assistant-powered smart speaker in their home. The results showed that families differ mainly in parents’ digital literacy skills, frequency of VA use, trust in technology, and preferred degree of child media mediation. Parents’ motivation for using VAs is primarily driven by enjoyment, especially when they use the VA together with their children. The study suggests finding new ways to guide the use of technology in families. Developers should focus on making VA use enjoyable for families, while scholars and policymakers should consider additional intervention criteria for family VA-use practices in the future (Wald et al., 2023). Another study by Carvalho et al. (2015) unveiled that Information Communication and Technology (ICT) brought about qualitative changes in family functioning. The most significant impact was explored on adolescents due to their frequent exposure to phones, social media, and larger networking sites leading to higher levels of cortisol awakening response (CAR) which was apparently among the fathers as compared to mothers due to their frequent use of mobile phones and emails (Afifi et al., 2018).

In contrast, the study by Gong et al. (2021) documented that there was a statistically significant relationship between family well-being and personal happiness and having more groups, receiving/sending photos/pictures, video calls, and instant messaging (Gong et al., 2021). However, the mobile usage of parents and children has increased their alone-time and mobile phone use as compared to the levels in 2015 (Mullan and Chatzitheochari, 2019). Since parents know about privacy and safety concerns using online tools and other technology, they do not favor their children’s active participation in online pedagogical activities such as building games, simulators, and puzzles (Brito and Dias, 2020). Nevertheless, it has been substantiated by the research that the personal use of ICT has a diminishing impact on the quality of relationships either parenting or romantic (Tammisalo and Rotkirch, 2022).

**Figure 1:**
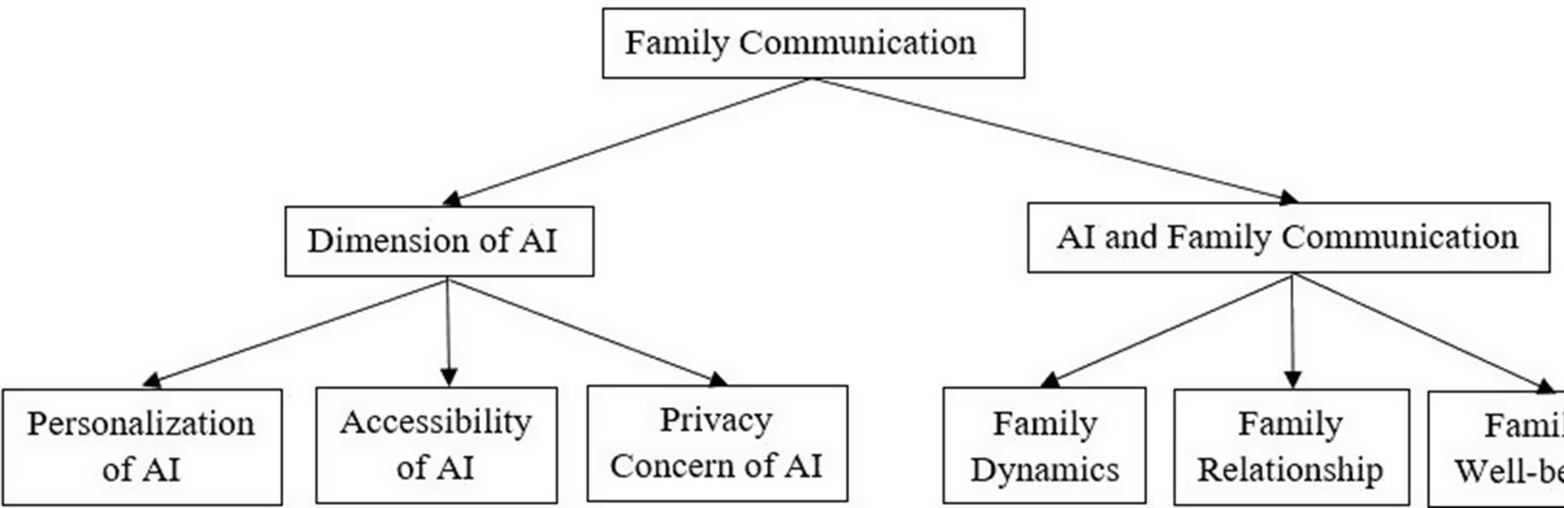
Theoretical framework.

Therefore, in the present study, the way different dimensions of AI impact the family dimensions will be studied. The study findings would generate new insights and data related to the impact of AI on family communication. The results of the study would add up to the existing body of literature on communication, and family dynamics. This research discovers the intersection of AI technology, psychology, sociology, and ethics within the family context. By addressing ethical considerations such as privacy and emotional well-being within the family, this research contributes to the growing body of literature on AI ethics, particularly about domestic life. Despite AI’s pervasive influence on daily life, there is a notable lack of research on its impact on family communication. This study addresses this gap by examining how AI dimensions; accessibility, personalization, language translation, privacy, bias, dependence, and safety–affect family dynamics. Understanding these impacts is crucial as AI becomes more integrated into daily interactions. Family communication is foundational to relationships, emotional well-being, and social development. By investigating AI’s influence, this study aims to enhance relationships, uncover potential risks such as privacy concerns and dependency, and provide evidence-based recommendations for responsible AI use. Filling this research gap will contribute to the well-being of families and guide the thoughtful integration of AI technologies into family life. Based on the study gap following theoretical framework is given below (Figure 1).

## 3 Methodology

### 3.1 Study design

The study employs a quantitative study approach to answer the research objective. A cross-sectional study design was adopted. Cross-sectional studies’ surveys are useful to investigate different phenomena when it comes to assessing disease prevalence, knowledge levels, and attitudes, and to validate for a comparison (Kesmodel, 2018). Capturing data from a diverse sample of participants at a single point in time provides an overview of how variables are distributed within the population. This approach is efficient and cost-effective, making it suitable for studies with limited resources or time constraints. Moreover, it allows researchers to explore relationships between variables and assess prevalence or correlations without the need for long-term follow-up. These characteristics make the cross-sectional design particularly valuable for generating quick insights into the current status of phenomena within a population, which can inform further research or interventions effectively.

The study design is helpful to document and assess the current perceptions of the participants as AI has been massively used in communication everywhere from homes to professional settings, in particular after the launch of Chatgpt. This approach helps explore how families perceive AI due to its efficiency, providing a timely snapshot of current attitudes across a diverse range of demographics and comparing perceptions among different groups which add to the identification of variations based on factors such as socioeconomic status or cultural background. We included a popup message within the questionnaire that provided examples of AI tools, such as language translation software, image enhancement tools, video editing software, voice assistants, and ChatGPT. This was done to ensure that all respondents were familiar with AI and its applications before completing the survey. Another rationale to adopt this study design was the ease of analysis and quick turnaround time which makes it suitable for informing policymakers promptly about emerging concerns related to AI.

### 3.2 Sampling technique

The participants of the study were recruited through social media, email newsletters, and family-oriented forums. A convenience sampling technique was adopted to recruit the study participants based on factors such as gender, age, and the number of AI tools. This sampling technique assists in preventing many of the limitations associated with research. For instance, using friends or family in the study is easier than targeting unknown individuals (Taherdoost, 2016).

### 3.3 Study participants

An online sample size calculator, Raosoft was used to determine the sample size of the study. The recommended sample size was 220. However, the researcher recruited 300 participants. The survey was conducted from November 1st, 2023 to February 29, 2024.

### 3.4 Study tool

A closed-ended survey was administered to conduct an online survey, the participants were approached via social media, email newsletters, and family-oriented forums. They were sent the link to the survey through these platforms. The researcher reviewed the published literature and formulated the questionnaire based on the seven aforesaid dimensions. The questionnaire comprised six sections namely, accessibility, personalization, language translation, privacy and bias concerns, dependency, and safety, with demographic details (Appendix A). The participants were instructed to mention the number of AI devices they had been using for language translation, image enhancing, video enhancing, videos and image editing, voice assistants, and Chatgpt for texting in a popup message in the online questionnaire to make them familiar with the AI tools.

### 3.5 Pilot test

A pilot test was conducted before conducting the study to examine the clarity and reliability of the survey questionnaire. A small sample of 30 participants was involved in the pilot test which was representative of the larger study population, to ensure that the questions were understood as intended and that the survey format was user-friendly. Feedback from the pilot participants led to minor revisions in the wording of several questions to enhance clarity. The reliability of the questionnaire was assessed using Cronbach’s Alpha, with a value exceeding 0.7, confirming that the instrument was sufficiently reliable for the full-scale study. This preliminary testing helped to refine the survey tool and provided confidence in its use for collecting accurate and consistent data in the main study (Table 1).

**Table 1:** Reliability statistics.

	Cronbach’s Alpha	N of items
Accessibility	0.721	2
Personalization	0.754	3
Language translation	0.784	3
Privacy and bias concerns	0.721	4
Dependency and safety	0.771	5
Total	0.748	18

### 3.6 Data collection

The data was collected through a survey questionnaire, and the responses of the participants were entered into a spreadsheet. The questionnaire consisted of 18 items in six sections, measured using a five-point Likert scale, encompassing Strongly Disagree, Disagree, Neutral, Agree, and Strongly Agree, respectively. The survey questionnaire was administered by sending e-mail invitations, which comprised an overview of the research, the tentative time to complete it, and the link to the questionnaire. Two reminder emails were sent to encourage participation. During the first week, the first reminder and in the third week, another reminder was sent. The researcher received 358 responses. A total of 300 responses were considered for data analysis as the rest of the survey responses were discarded due to incomplete answers.

### 3.7 Data analysis

For data analysis, the Statistical Package for the Social Sciences (SPSS) version 27.0 was used to perform descriptive statistics to know the extent of use of AI devices in family communication, and the age groups, most prefer to use AI in family. A multiple regression model was performed to examine the impact of different AI dimensions on family communication.

### 3.8 Ethical considerations

Ethical consideration were made while conducting the study. Along with the online survey form, informed consent was obtained from the participants before starting the study. All the participants were briefed about the research aims, and confidentiality of their identity, and only the intended use of the collected data. The current study prioritizes comprehensive data protection measures to safeguard participant confidentiality and anonymity with the help of transparently communicating these protocols and ensuring adherence to ethical guidelines.

### 3.9 Results

Table 2 displays the demographic details of the participants such as age, gender, and number of AI devices used for language translation, image enhancing, video enhancing, videos and image editing, voice assistants, and Chatgpt for texting. The majority of the participants were males, aged between 31 and 35 years and most of them (110) had been using more than five AI tools.

**Table 2:** Demographic details.

	Frequency	Percent
Age		
25–30	93	31.0
31–35	111	37.0
36–40	96	32.0
Gender		
Female	148	49.3
Male	152	50.7
The number of AI tools usage		
1	2	0.7
2	1	0.3
4	187	62.3
5	110	36.7

Table 3 displays the questionnaire outcomes which provide an overview of how AI tools impact family communication across various aspects. With respect to accessibility, the majority of respondents (98%) agree that AI tools have significantly increased access to communication. However, when it comes to assisting family members with disabilities, the responses are more diverse; while a slight majority (67%) agree that AI tools have been helpful. In personalization, 58% of respondents believe that AI provides the best-customized responses for family communication, a significant minority (16%) disagree, suggesting that personalization may not meet everyone’s expectations. A larger majority (76%) agree that AI devices introduce innovative features that shape new aspects of communication.

**Table 3:** Descriptive statistics.

Questions	Strongly disagree	Disagree	Neutral	Agree	Strongly agree
Accessibility					
AI tools have increased our access to communication.	1 (0.3%)	1 (0.3%)	0 (0.0%)	14 (4.7%)	284 (94.7%)
AI devices have been improving our family communication by helping our members with disabilities (such as speech impairment and difficulty in recognition).	0 (0.0%)	2 (0.7%)	89 (29.7%)	117 (39.0%)	92 (30.7%)
Personalization					
AI tools provide the best customized responses to our communication in the family.	14 (4.7%)	2 (0.7%)	90 (30.0%)	98 (32.7%)	96 (32.0%)
AI devices shape new aspects of our communication with innovative features.	1 (0.3%)	0 (0.0%)	108 (36.0%)	93 (31.0%)	98 (32.7%)
AI is paired with personalized communication which helps us better understand our expectations for communication and the exchange of ideas in different formats and stylistic versions.	13 (4.3%)	14 (4.7%)	101 (33.7%)	73 (24.3%)	99 (33.0%)
Language translation					
AI has reduced most of the barriers to communication than ever before in the form of providing over-the-counter translation.	1 (0.3%)	0 (0.0%)	105 (35.0%)	71 (23.7%)	123 (41.0%)
AI-based spoken translation, available in all languages and accents is an incredibly useful feature.	1 (0.3%)	1 (0.3%)	77 (25.7%)	114 (38.0%)	107 (35.7%)
AI tools provide high-quality text translations, improving cross-lingual communication.	0 (0.0%)	2 (0.7%)	79 (26.3%)	125 (41.7%)	94 (31.3%)
Privacy and bias concerns					
We are concerned about the data and privacy of communication of our family.	39 (13.0%)	19 (6.3%)	72 (24.0%)	87 (29.0%)	83 (27.7%)
We do not know the AI privacy features to protect our data and communication.	0 (0.0%)	27 (9.0%)	87 (29.0%)	99 (33.0%)	87 (29.0%)
We have encountered some algorithm bias while using the features of AI in family communication.	0 (0.0%)	1 (0.3%)	115 (38.3%)	94 (31.3%)	90 (30.0%)
We are concerned about the usage of our data and communication amid the rising analysis of personal information by AI to new levels of power and speed.	1 (0.3%)	0 (0.0%)	106 (35.3%)	73 (24.3%)	120 (40.0%)
Dependency and safety					
Our families are increasingly depending on AI tools to respond to communication.	0 (0.0%)	1 (0.3%)	82 (27.3%)	87 (29.0%)	130 (43.3%)
We are sure about the safety features and encryption of AI systems and the data they use.	11 (3.7%)	1 (0.3%)	76 (25.3%)	108 (36.0%)	104 (34.7%)
The use of AI devices has increased our dependence on decision-making. and overall impact and prospects.	1 (0.3%)	1 (0.3%)	112 (37.3%)	72 (24.0%)	114 (38.0%)
AI devices provide smart replies and suggested feedback and answers which save time.	0 (0.0%)	1 (0.3%)	109 (36.3%)	95 (31.7%)	95 (31.7%)
AI devices have increased the speed of communication and improved interpersonal perceptions in families.	0 (0.0%)	9 (3.0%)	95 (31.7%)	100 (33.3%)	96 (32.0%)
Overall impact					
The impact of AI is positive on family communication.	1 (0.3%)	1 (0.3%)	68 (22.7%)	92 (30.7%)	138 (46.0%)

Regarding language translation, most respondents feel positive, with a majority agreeing that AI has reduced communication barriers and improved cross-lingual communication through high-quality translations. However, there is considerable concern about data privacy and algorithmic bias, with a substantial number expressing unease. In the area of dependency and safety, the answers to the questionnaire revealed an increasing reliance on AI tools for communication, with many respondents (77%) confident in the safety features and encryption of AI systems. The overall impact of AI on family communication is perceived positively, with a majority (69%) agreeing that AI has had a beneficial influence.

The impact of accessibility on family communication has been measured through a linear regression model (Table 4). The results obtained from the regression model run on SPSS, depict predictions that are almost nearing the actual values to predict the impact. The results of the regression model suggest that accessibility (*p* = 0.001), personalization (*p* = 0.001), and language translation (*p* = 0.016) had a statistically significant impact on family communication.

**Table 4:** Model summary.

Model	*R*	*R* square	Std. Error of the estimate	Sig.
Accessibility				
1	0.784	0.534	5.501	0.001
Personalization				
2	0.745	0.560	0.556	0.001
Language translation				
3	0.784	0.534	5.501	0.016
Dependency and safety				
4	0.759a	0.529	0.536	0.001
Overall impact				
5	0.756a	0.526	0.537	0.001

The R represents the value of R, the multiple correlation coefficient. It is an indicator of the quality of the prediction of the dependent variable. In this case, accessibility (0.784), personalization (0.745), and language translation (0.784) show a good level of prediction. The results in Table 5 present the statistical model of multiple regression for the impact of AI tools usage on accessibility, personalization, and language translation in the domain of family communication. These variables statistically significantly predicted the relationship and added statistical significance to the prediction, *p* < 0.05.

**Table 5:** ANOVA for gender-wise impact on family communication.

		df	Mean Square	*F*	Sig.
Accessibility	Between groups	2	1.135	5.230	0.006
	Within groups	297	0.217		
Personalization	Between groups	2	0.775	2.090	0.126
	Within groups	297	0.371		
Language translation	Between groups	2	1.165	4.655	0.010
	Within groups	297	0.250		
Dependency and safety	Between groups	2	1.125	4.365	0.004
	Within groups	297	0.356		
Overall impact	Between groups	2	0.864	5.126	0.003
	Within groups	297	0.264		

To find whether the impact of AI dimensions such as accessibility, personalization, and language translation varies differently based on gender, One-way Analysis of Variance (ANOVA) was performed (Table 5). The results showed that in terms of personalization, there was no difference in terms of impacting family communication (*p* = 0.126). Whereas, accessibility (*p* = 0.006), language translation (*p* = 0.010), dependency and safety (*p* = 0.004) and overall impact (*p* = 0.003).

To assess whether there was any association between the number of AI tools on privacy and bias concerns, dependency and safety concerns, the findings in Table 6 reveal that using multiple AI tools was statistically associated with raising concerns about bias and privacy (*p* = 0.015), safety and dependence (*p* = 0.049) of parents. These results provide significant insights into the different impacts of different AI dimensions on family communication. On one hand, using AI tools helps to provide greater means of accessing AI chatbots while providing exciting and generative features to respond to and communicate with others. On the other hand, parents perceived that having dependence on AI led to a greater likelihood of experiencing bias, and a greater risk of safety, and privacy. Also, AI tools are perceived as the easiest tools to use for translation. However, due to using AI voice assistant, there was no significant impact on family communication in light of the results. It implies that maximizing the vitality of AI tools alone without addressing the increasing concerns for privacy bias, and safety, will increase the risk of using AI tools in family communication.

**Table 6:** Impact of the number of AI tools.

		Sum of Squares	df	Mean Square	F	Sig.
Bias and privacy	Between groups	18.985	4	4.746	21.050	0.015
	Within groups	66.514	295	0.225		
	Total	85.499	299			
Safety and dependence	Between groups	10.664	4	2.666	21.081	0.049
	Within groups	37.306	295	0.126		
	Total	47.969	299			

## 4 Discussion

The study presents an analysis of the way different dimensions of AI impact family communication and reveals that these dimensions such as language translation, personalization, and accessibility led statistically significant impact on family communication. These findings are similar to the results of the study by Hohenstein et al. (2023) which concluded that AI algorithms brought about greater changes in the way of responses and social relationships.

The findings of the present study revealed that around 62% of people believed that it has increased our dependence on decision-making and overall impact and prospects which also aligned with the results study conducted by Ahmad et al. (2023), aiming to scrutinize the impact of AI on decision-making, and laziness. The study concluded that AI exacerbated laziness among humans and suggested taking on-time and pragmatic measures before instilling AI devices to stave off such elements. Moreover, Goldenthal et al. (2021) examined six functional AI-mediated communications (AI-MC) tools, such as voice-assisted communication, language correction, predictive text suggestion, transcription, translation, and personalized language learning. However, the findings of the study are contrastive to the present study based on the survey findings with 519 participants where it was found that there is no one-size-fits-all strategy for communication in different groups (Goldenthal et al., 2021).

Likewise, in the present study, another study has highlighted that there is a need to create a trustworthy AI (Vincent-Lancrin and Van der Vlies, 2020). In addition, Shneiderman (2020) recommended raising the safety, reliability, and trustworthiness of human-centric AI systems, safety culture, and trustworthy certification with the help of independent oversight. Galaz et al. (2021) have further recommended addressing systematic risks, associated with AI communication networks and algorithm bias.

In addition, the study found a gender-wise impact on family communication (*p* < 0.05) except personalization where in terms of personalization, there was no difference in terms of impacting family communication (*p* > 0.05). These findings are different from the findings of Straw and Callison-Burch (2020) who reviewed and analyzed 52 studies and found gender bias in clinical data, data science, and linguistic perspective which is reflected in the AI algorithm called algorithm bias. Leavy (2018) stressed ensuring gender balance to stave off algorithm bias which perpetuates disadvantage to women in AI-driven tools.

The study’s outcomes accurately reflect a notable reduction in communication time attributed to the use of AI tools within family dynamics. This reduction is observed to correlate with changes in the frequency and nature of positive emotional language used among family members (63.3%). Specifically, AI tools streamline communication processes, allowing for quicker information exchange and task completion, which in turn may influence the tone and emotional dynamics of interactions (65.3%).

The study underscores that while AI facilitates efficiency in communication, its impact on the use of positive emotional language varies and requires nuanced exploration across different family contexts and interaction patterns. De Lima et al. (2023) underscored that mostly the AI models are impacted by societal, technical, and individual bias in the technical and societal perspective which can be staved off through debiasing, and inculcating gender sensitivity (De Lima et al. (2023), fairness in AI development (Nadeem et al., 2020) and opting a feminist lens and affective labor concept (Manasi et al., 2022). These results are similar to the study by Zhai and Wibowo (2023) who studied the efficacy of AI based on six dimensions which were technological integration, task designs, students’ engagement, learning objectives, technological limitations, and the novelty effect. The study documented the effectiveness of the AI dialog system but suggested introducing features related to culture, empathy, and humor to provide a realistic learners’ experience. It asserted that the adoption of the AI system in communication and language acquisition in Saudi Arabia was in its initial stage (Zhai and Wibowo, 2023).

The results of Urlaub and Dessein (2022) are dissimilar to the present study as it pinpointed the probability of bias and concerns related to transparency, and fairness as a result of the advent of more advanced and capable translation assistants dealing with multilingual translation tasks despite their significance. They indicated the overreliance on the technology of machine translation without considering the limitations of machines in handling cultural and linguistic complexities (Urlaub and Dessein, 2022). Therefore, sensitization is needed to be familiar with the limitations of technology, the richness of human interaction in an authentic way, and humanistic communication (Huang, 2022) with a balanced and blended approach to human creativity, critical thinking, and AI (Jiang, 2022). Beyond family communication, Murero et al. (2020), documented the significance of Voiceitt® which is used for non-standard speech recognition. This tool served as the Augmentative Alternative Communication (AAC) technology to be used for people having speech impairments which has been initiating ease and quality in the lives and services of patients, their caregivers, and societies (Murero et al., 2020).

### 4.1 Strengths and limitations

The contribution of this study lies in updating the communication literature about AI usage in family communication. The paper adopts an unconventional and more nuanced approach to assess different dimensions of AI independently and their relevance in communication taking place in a family. It provided the results related to different dimensions of AI and the way they affect it. Therefore, this serves as a novel attempt to explore the impact of different AI dimensions on family communication. It provides novel insights for developers, researchers, field experts, and decision-makers to scrutinize the role of different dimensions of AI in family communication. The emphasis on the necessity of AI in the practical implementation of technology development highlights its role in enhancing efficiency, accessibility, and personalization in various domains, including family communication. By addressing specific needs and challenges within family dynamics, AI can offer valuable tools that streamline tasks, improve accessibility for all members, and customize interactions based on individual preferences. However, careful consideration of ethical implications, privacy concerns, and potential biases is crucial in developing AI technologies that responsibly integrate into family life. Balancing the benefits of AI with these considerations ensures that its implementation aligns with the diverse needs and values of families while promoting positive outcomes in communication and interaction.

However, the study got its empirical findings through survey observations not supported by interviews which serves as its limitations. Furthermore, the use of convenience sampling via social media and family-oriented forums introduces selection bias, limiting the sample’s diversity and generalizability. This recruitment method may result in a biased sample, as participants from these platforms may have different attitudes and experiences with AI compared to those who do not engage with these platforms, thereby affecting the study’s external validity.

### 4.2 Future researches

Since studying the dimensions of AI is a new aspect of research, there is a huge potential for researchers to explore other phenomena in the domain of family communication. Future studies can be conducted with a greater sample size with a mixed-method study design. Moreover, employing a mixed-method approach, i.e., surveys and interviews can add further interesting and useful insights to the literature on family communication with the integration of AI.

Additionally, advanced analysis techniques like Partial Least Squares (PLS) or Structural Equation Modeling (SEM) with AMOS are recommended over SPSS for more robust insights. These methods offer superior capabilities for path analysis, moderation, and mediation analysis, which are crucial for understanding complex relationships among variables. Employing these techniques will enhance the reliability and validity of constructs related to AI usage and provide deeper insights into the impact of AI on family communication.

Further, in future research, the longitudinal study should be designed to assess changes in attitudes and behaviors over time, providing a more comprehensive understanding of the long-term impact of AI on family communication dynamics.

## 5 Conclusion

This study presents empirical findings highlighting the positive impact of various dimensions of AI on family communication. The research reveals that AI technologies have significantly enhanced communication effectiveness and interpersonal perceptions among family members since their adoption. However, it also identifies critical policy concerns regarding data protection and storage associated with AI devices. These concerns stem from insufficient knowledge about privacy policies and a lack of transparency in related mechanisms. Moreover, the study uncovers a growing dependency among family members on AI devices, driven by their reliance on the automatic features offered by these tools.

## Data Availability

The raw data supporting the conclusions of this article will be made available by the authors, without undue reservation.
