# Supplementary material for: Dimensions of artificial intelligence on family communication
Source: Front Artif Intell. 2024 Sep 11;7:1398960. doi: 10.3389/frai.2024.1398960 (PMC11422382; doi:10.3389/frai.2024.1398960)
Supplement: Supplementary file 1 [file Data_Sheet_1.pdf]

Appendix A

Study Questionnaire

Demographic Information

Age:

- **25-30**
- **31-35**
- **36-40**

Gender: Male/Female

Ownership of AI devices

- **Yes**
- **No**

A. Accessibility

A1. AI tools have increased our access to communication.

Strongly Disagree 1	Disagree 2	Neither Agree nor Disagree 3	Agree 4	Strongly Agree 5

A2. AI devices have been improving our family communication by helping our members with disabilities (such as speech impairment and difficulty in recognition).

Strongly Disagree	Disagree	Neither Agree nor Disagree	Agree	Strongly Agree

P. Personalization

P1. AI tools provide the best customized responses to our communication in the family.

Strongly Disagree	Disagree	Neither Agree nor Disagree	Agree	Strongly Agree

P2. AI devices shape new aspects of our communication with innovative features.

Strongly Disagree	Disagree	Neither Agree nor Disagree	Agree	Strongly Agree

P3. AI is paired with personalized communication which helps us better understand our expectations for communication and the exchange of ideas in different formats and stylistic versions.

Strongly Disagree	Disagree	Neither Agree nor Disagree	Agree	Strongly Agree

LT. Language Translation

LT1. AI has reduced most of the barriers to communication than ever before in the form of providing over-the-counter translation.

Strongly Disagree	Disagree	Neither Agree nor Disagree	Agree	Strongly Agree

LT2. AI-based spoken translation, available in all languages and accents is an incredibly useful feature.

Strongly Disagree	Disagree	Neither Agree nor Disagree	Agree	Strongly Agree

LT3. AI tools provide high-quality text translations, improving cross-lingual communication.

Strongly Disagree	Disagree	Neither Agree nor Disagree	Agree	Strongly Agree

BP. Privacy and Bias Concerns

BP1. We are concerned about the data and privacy of communication of our family.

Strongly Disagree	Disagree	Neither Agree nor Disagree	Agree	Strongly Agree

BP2. We do not know the AI privacy features to protect our data and communication.

Strongly Disagree	Disagree	Neither Agree nor Disagree	Agree	Strongly Agree

BP3. We have encountered some algorithm bias while using the features of AI in family communication.

Strongly Disagree	Disagree	Neither Agree nor Disagree	Agree	Strongly Agree

BP4. We are concerned about the usage of our data and communication amid the rising analysis of personal information by AI to new levels of power and speed.

Strongly Disagree	Disagree	Neither Agree nor Disagree	Agree	Strongly Agree

DS. Dependency And Safety

DS1. Our families are increasingly depending on AI tools to respond to communication.

Strongly Disagree	Disagree	Neither Agree nor Disagree	Agree	Strongly Agree

DS2. We are sure about the safety features and encryption of AI systems and the data they use.

Strongly Disagree	Disagree	Neither Agree nor Disagree	Agree	Strongly Agree

DS3. The use of AI devices has increased our dependence on decision-making.

and overall impact and prospects.

Strongly Disagree	Disagree	Neither Agree nor Disagree	Agree	Strongly Agree

DS4. AI devices provide smart replies and suggested feedback and answers which save time.

Strongly Disagree	Disagree	Neither Agree nor Disagree	Agree	Strongly Agree

DS5. AI devices have increased the speed of communication and improved interpersonal perceptions in families.

Strongly Disagree	Disagree	Neither Agree nor Disagree	Agree	Strongly Agree

IM. Overall Impact

IM1. The impact of AI is positive on family communication.

Strongly Disagree	Disagree	Neither Agree nor Disagree	Agree	Strongly Agree

<Thank you for participation>
